# Prevalence of complications and comorbidities associated with obesity: a health insurance claims analysis

**DOI:** 10.1186/s12889-024-21061-z

**Published:** 2025-01-22

**Authors:** Jay P. Bae, David R. Nelson, Kristina S. Boye, Kieren J. Mather

**Affiliations:** Eli Lilly and Company, Indianapolis, IN 46285 USA

**Keywords:** Obesity, Comorbidities, Hypertension, Dyslipidemia, Cardiovascular diseases

## Abstract

**Background:**

Despite the substantial burden of obesity in the United States (US), data on the comprehensive range of comorbidities in different age groups is limited. This study assessed the prevalence of various comorbidities among people diagnosed with obesity (as per ICD-10 diagnosis code) across age cohorts and compared how they differ from people without obesity.

**Methods:**

This cross-sectional study analyzed individuals from all four regions (Midwest, Northeast, South, and West) of the US who had continuous insurance coverage from 2018 to 2020, using a large health insurance claims database (Merative™ MarketScan^®^). Identification of disorders relied on ICD-10 diagnosis code in patient claims and their prevalence was calculated.

**Results:**

Of 6,935,911 individuals, people with a diagnosis of obesity accounted for 22.0%, 33.6%, and 34.4% in the 18–39 years, 40–64 years, and ≥ 65 years age groups, respectively. Within age strata, the mean age of people with obesity was comparable with those without obesity. Comorbidity burden was significantly higher among people with obesity, but increased with age in both obesity and non-obesity groups. Comorbidities with highest prevalence in people with obesity included: (i) hypertension (18–39 years: 29.0%, 40–64 years: 66.2%, ≥ 65 years: 89.4%), (ii) dyslipidemia (18–39 years: 28.1%, 40–64 years: 65.4%, ≥ 65 years: 88.0%), (iii) depression or anxiety (18–39 years: 44.1%, 40–64 years: 39.0%, ≥ 65 years: 38.9%), and (iv) prediabetes (18–39 years: 17.1%, 40–64 years: 32.2%, ≥ 65 years: 35.3%). Notably, increased prevalence of cardiometabolic risk factors such as hypertension and dyslipidemia began at an earlier age in people with obesity as compared with those without obesity. Ratio of prevalence between obesity and non-obesity groups was highest for the 18–39 years age group, as compared to older groups. Disorders such as obstructive sleep apnea, osteoarthritis, type 2 diabetes, metabolic dysfunction-associated steatotic liver disease, coronary heart diseases (CHD), and chronic kidney diseases also exhibited substantial burden among those with obesity.

**Conclusions:**

In this claims study, hypertension and dyslipidemia were the leading comorbidities in people with obesity, with an increasing prevalence with age. The burden of cardiometabolic comorbidities among the younger age group suggested potential risk for early onset of CHD in later life. Understanding the range of obesity-related comorbidities seen in this claims data may encourage healthcare professionals and healthcare systems to systematically diagnose and better manage these disorders. Further research using additional data sources can offer a more accurate view of the prevalence of obesity and its impact.

## Introduction

Obesity has reached pandemic proportions globally and is a major public health challenge. The prevalence of obesity in the United States (US) was approximately 42% from 2017 to 2020 [1]. Studies have estimated that nearly one in two adults will have obesity in the US by 2030 [2]. People with obesity have reduced health-related quality of life compared with individuals of normal weight [3, 4]. In addition, obesity imparts social disadvantages and contributes to reduced economic productivity [5]. In the US, obesity is associated with excess medical costs, accounting for $172.74 billion of annual expenditures [6]. Obesity is a complex, multicomponent metabolic disease which contributes to numerous disorders such as hypertension, dyslipidemia, type 2 diabetes, osteoarthritis, cardiovascular diseases, cancer, fatty liver disease, myocardial infarction, stroke, dementia, and obstructive sleep apnea [7, 8]. Research shows that obesity is also associated with higher all-cause mortality, with life expectancy estimated to be shortened by three to four years in people with obesity compared with normal weight individuals [9, 10].

One of the important aspects of obesity-related health burden is a significant increase in early onset of obesity among young adults [11]. A serial cross-sectional study encompassing data from 2009 to March 2020 from the US reported an increase in the prevalence of obesity (from 32.7 to 40.9%) among young adults aged 20 to 44 years [12]. Notably, the onset of obesity at a young age is associated with a greater risk of worsening obesity over time than at an older age [13].

Despite the high prevalence of obesity, the rates of diagnosis remain low [14]. The under-diagnosis of obesity may be driven by numerous factors, including the prioritization of obesity-related complications over obesity during healthcare visits, or challenges with ICD coding and reimbursement for obesity care. Understanding the comprehensive range of comorbidities of obesity in different age groups is also important for their timely identification and management. Better recognition of obesity-related comorbidities may encourage healthcare professionals and healthcare systems to systematically diagnose and manage obesity. Hence, this study assessed the prevalence of comorbidities among individuals with obesity across age groups in the real-world setting, and compared how they differ from those without obesity.

## Methods

### Study design

This retrospective, observational study was conducted using health insurance claims data from the Merative™ MarketScan^®^ Research Databases (Commercial Claims and Encounters and the Medicare Supplemental and Coordination of Benefits). The Commercial Database contains the inpatient, outpatient, and outpatient prescription-drug experience of employees and their dependents, covered under a variety of health plans, including exclusive provider organizations, preferred provider organizations, consumer-driven health plans, and high-deductible health plans. A detailed description of the database is provided on the official website (https://www.merative.com/documents/brief/marketscan-explainer-general). The Medicare Supplemental Database contains the same information for individuals with Medicare supplemental insurance paid for by employers. The study included data from individuals from all four US Census regions (Midwest, Northeast, South, and West) with varying proportions of the population. However, this study did not include individuals who were uninsured or were receiving other types of health insurance, such as Medicaid.

### Study population

All individuals with continuous insurance coverage from January 2018 to December 2020 were included in the study. Additional data from the post International Classification of Diseases, 10th Revision, Clinical Modification (ICD-10-CM) era of October 2015 to December 2017, if available, were used to capture additional medical history of the patients.

### Outcomes

Individuals included for data analysis were classified into obesity and non-obesity groups based on presence of obesity-related comorbidities identified by ICD-10-CM diagnosis codes. Identification of disorders also relied on ICD-10-CM diagnosis codes in patient claims and their prevalence was calculated. The current study used obesity-related codes in ICD-10: E66.x except 66.3, and Z codes specific to body mass index (BMI) (Z68.3x and Z68.4x). Based on the current literature, the following disorders whose prevalence is associated with obesity were selected for comparative analysis: (i) chronic kidney disease/ diabetic kidney disease, (ii) coronary heart disease, (iii) depression/anxiety, (iv) dyslipidemia/ hyperlipidemia, (v) heart failure, (vi) hypertension, (vii) malignancies, (viii) metabolic dysfunction-associated steatotic liver disease/ metabolic dysfunction-associated steatohepatitis/ liver cirrhosis, (ix) obstructive sleep apnea, (x) osteoarthritis, xi) polycystic ovarian syndrome (females only), xii) prediabetes, xiii) type 2 diabetes, and xiv) osteoporosis, which may have a negative association with obesity. Individual patients with corresponding diagnoses were classified into the respective disorder groups. Prediabetes and type 2 diabetes were assessed as mutually exclusive criteria; when both diagnoses were present, the patient was assigned to the type 2 diabetes group. The patient’s age was set as of January 1, 2018.

### Compliance with ethics guidelines

All study data were analyzed per protocol and compliant with the US patient confidentiality requirements, including the Health Insurance Portability and Accountability Act (HIPAA) of 1996 regulations. As all databases used in the study were fully de-identified and compliant with the HIPAA, the study was exempt from Institutional Review Board approval.

### Statistical analyses

The study population was stratified into three age groups (18–39, 40–64, and ≥ 65 years) and data were compared for people with and without obesity. Descriptive statistics were used to describe the demographic characteristics including age, sex, insurance plan type, and US region. We compared the prevalence rates of different obesity-related comorbidities among people with and without obesity by age group. Comorbidity count by age groups was assessed using mean and relative ratio among people with and without obesity. Descriptive data was presented as mean (standard deviation [SD]) for continuous variables and percentages for categorical variables. The proportion of patients with a qualifying diagnosis was reported as crude prevalence. Chi-square tests were used for categorical variables, and Wilcoxon rank-sum tests were applied to continuous and ordinal variables. A two-sided p-value < 0.05 was considered significant. SAS version 9.4 (SAS Institute, Inc, Cary NC) was used for data analysis.

## Results

### Demographic characteristics

A total of 6,935,911 people were identified for the analysis (age groups: 18–39 years: 38.1%, 40–64 years: 57.5%, and ≥ 65 years: 4.4%). Regional sample distribution by obesity status is provided in Table 1. People with a diagnosis of obesity accounted for 22.0%, 33.6%, and 34.4% in the 18–39 years, 40–64 years, and ≥ 65 years age groups, respectively. The mean age of people with obesity was comparable with those without obesity across the age groups (within 2.5 years). The proportion of females with obesity was higher than males across the ages, except for the ≥ 65 years age group. In the 18–39 years and 40–64 years age groups, the prevalence of obesity was highest in the South census region, followed by the Midwest, Northeast, and West. However, in the ≥ 65 years age group, obesity prevalence was highest in the Midwest census region, followed by the South, Northeast, and West regions.

**Table 1:** Demographic characteristics among people with and without obesity by age cohort

Demographic characteristic (%), (*N* = 6,935,911)	18–39 Years			40–64 Years			≥ 65 Years		
	Obesity	Non-obesity	Total	Obesity	Non-obesity	Total	Obesity	Non-obesity	Total
**N (%)**	581,065 (8.4%)	2,060,085 (29.7%)		1,342,453 (19.4%)	2,647,918 (38.2%)		104,655 (1.5%)	199,735 (2.9%)	**6**,**935**,**911** 100%
**Age at index date, Mean (SD)**	30.67 (6.32)	28.25 (6.89)		51.42 (6.33)	51.12 (6.41)		72.60 (5.98)	74.44 (7.29)	
**Sex (% obesity/% non-obesity)**									
Male	17.5	82.5	100%	31.5	68.5	100%	34.7	65.3	100%
Female	26.4	73.6	100%	35.6	64.4	100%	34.1	65.9	100%
**Insurance plan type** ^**1**^ **(%)**									
Exclusive provider organization / Preferred provider organization	22.9	77.1	100%	34.3	65.7	100%	37.0	63.0	100%
Consumer-driven health plans / High-deductible health plan	20.4	79.5	100%	31.3	68.6	100%	17.0	83.0	100%
**Region (% based on diagnosis rate per population of each region)**									
Midwest	21.6	78.4	100%	34.4	65.6	100%	36.9	63.1	100%
Northeast	19.1	80.9	100%	30.4	69.7	100%	24.1	75.9	100%
South	24.9	75.1	100%	37.3	62.7	100%	30.0	70.0	100%
West	16.2	83.8	100%	23.9	76.1	100%	22.2	77.8	100%

*SD* standard deviation

Total number of people with obesity: 2,028,173; significance (*p* < 0.05) of obesity versus non-obesity for sex, insurance plan type, and region in the age groups: 18–39 years, 40–64 years, ≥ 65 years

^1^Other insurance plans represent 27.2% of the overall sample and are not shown, significance is based on all categories

### Prevalence of comorbidities in people with or without obesity across age groups

**Fig. 1:**
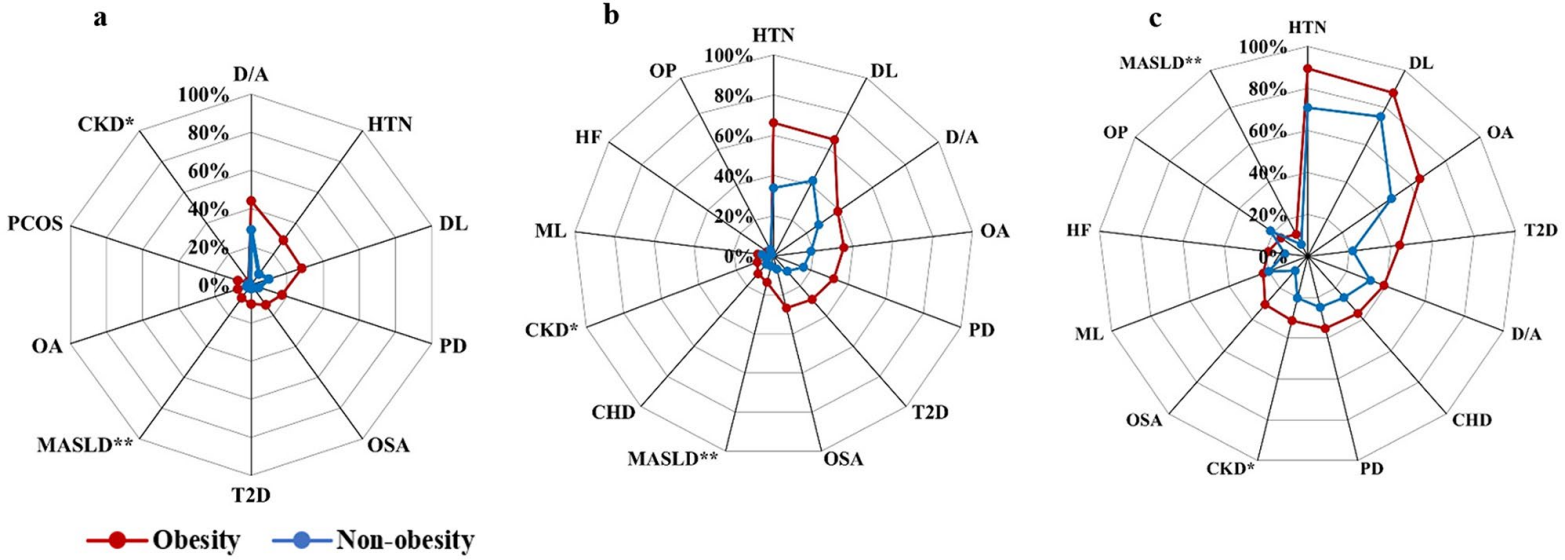
Overview of comorbidities prevalent among people with and without obesity by age group **a.** 18–39 years, **b**. 40–64 years, and **c**. ≥65 years. *CKD/ DKD, **MASLD/ MASH/ LC, *CHD* coronary heart disease, *CKD* chronic kidney disease, *D/A* depression/anxiety, *DKD* diabetic kidney disease, *DL* dyslipidemia, *HF* heart failure, *HTN* hypertension, *LC* liver cirrhosis, *ML* malignancies, *MASLD* metabolic dysfunction-associated steatotic liver disease, *MASH* metabolic dysfunction-associated steatohepatitis, *OSA* obstructive sleep apnea, *OA* osteoarthritis, *OP* osteoporosis, *PCOS* polycystic ovary syndrome, *PD* prediabetes, *T2D* type 2 diabetes. *p* < 0.001 for all comorbidities. Data presented in decreasing order of prevalence and only for comorbidities with a prevalence rate > 2%

In comparison to those without obesity, people with obesity had a significantly higher prevalence (*p* < 0.0001) of all the examined disorders across the ages. Except in the ≥ 65 years age group, where people without obesity had a significantly higher prevalence (*p* < 0.0001) of osteoporosis. In the younger group of 18–39 years, people with obesity had a higher burden of comorbidities versus those without obesity: depression/anxiety: 44.1% versus 28.9%, hypertension: 29.0% versus 7.1%, dyslipidemia: 28.1% versus 9.9%, prediabetes: 17.1% versus 4.4%, and obstructive sleep apnea: 13.0% versus 1.9% (Fig. 1). In the 40–64 years age group with or without obesity, hypertension and dyslipidemia were the most common comorbidities (Fig. 1). Notably, the prevalence of hypertension in the obesity group (66.2%) was nearly twice as much as in the non-obesity (33.8%) group. Dyslipidemia (65.4% versus 42.3%), depression/anxiety (39.0% versus 27.4%), osteoarthritis (35.3% versus 18.9%), and prediabetes (32.2% versus 15.9%) were other predominant comorbid disorders in the 40–64 years age group in the obesity and non-obesity groups. The older age group of ≥ 65 years with or without obesity had the highest burden of cardiometabolic disorders such as hypertension (89.4% and 70.8%), dyslipidemia (88.0% and 75.1%), and type 2 diabetes (44.3% and 21.8%), respectively. The older age group also showed a high prevalence of osteoarthritis (65.0% and 48.6%) and coronary heart diseases (36.2% and 26.1%) in both obesity and non-obesity groups, respectively.

We also studied the obesity-related comorbidities by age (Table 2). Hypertension and dyslipidemia had the highest prevalence beginning at a relatively early age in people with obesity, and the prevalence increased with age for both obesity and non-obesity groups. The burden of hypertension and dyslipidemia was substantially higher among the obesity versus non-obesity groups in the younger and middle age groups. However, the prevalence was comparable in the ≥ 65 years age group. Among people with obesity, the burden of depression was highest in the younger age group; however, the prevalence showed a minor decline and reached a plateau in the middle and older ages. People with obesity showed a comparatively higher prevalence of type 2 diabetes, which increased considerably with age. Notably, in the older age group, the prevalence of type 2 diabetes in the obesity group (44.3%) was nearly twice as much as in the non-obesity group (21.8%). In addition to cardiometabolic disorders, the prevalence of obstructive sleep apnea increased with age in the obesity group, from 13.0% in the 18–39 years age group to 26.6% in the 40–64 years age group. Similarly, among people with obesity, osteoarthritis showed a rapid increase in prevalence over the three age groups and became the third most prevalent disorder in the ≥ 65 years age group.

**Table 2:** Prevalence of comorbidities among people with and without obesity by age group (%)

Percentage	18–39 Years		40–64 Years		≥ 65 Years	
	Obesity	Non-obesity	Obesity	Non-obesity	Obesity	Non-obesity
Coronary heart disease	1.7	0.5	11.7	5.6	36.2	26.1
CKD/ DKD	2.4	0.8	8.8	3.6	31.4	20.4
Depression or anxiety	44.1	28.9	39.0	27.4	38.9	32.0
Dyslipidemia	28.1	9.9	65.4	42.3	88.0	75.1
Heart failure	0.8	0.2	4.0	1.2	19.0	11.0
Hypertension	29.0	7.1	66.2	33.8	89.4	70.8
Malignancies	1.9	1.1	8.0	5.9	22.6	19.8
MASLD/ MASH/liver cirrhosis	8.5	1.9	13.6	5.3	11.8	6.4
Osteoarthritis	7.4	2.5	35.3	18.9	65.0	48.6
Osteoporosis	0.2	0.2	2.7	3.7	15.5	21.6
Obstructive sleep apnea	13.0	1.9	26.6	6.6	30.6	9.1
Polycystic ovarian syndrome	7.4	1.6	1.1	0.3	0.1	0.0
Prediabetes	17.1	4.4	32.2	15.9	35.3	24.8
Type 2 diabetes	10.1	2.0	29.0	10.1	44.3	21.8

*CKD* chronic kidney disease; *DKD* diabetic kidney disease *MASLD* metabolic dysfunction-associated steatotic liver disease, *MASH* metabolic dysfunction-associated steatohepatitis

Significant (*p* < 0.0001) differences in obesity versus non-obesity were observed for all disorders in the groups 18–39 years, 40–64 years, and ≥ 65 years

Our study revealed that the mean comorbidity burden was significantly higher among people with obesity and increased with age in both obesity and non-obesity groups (Table 3). Notably, the relative ratios of comorbidities between obesity and non-obesity groups were highest for the 18–39 years group (2.73), as compared to older groups of 40–64 years (1.90) and ≥ 65 years (1.36; Table 3).

**Table 3:** Comorbidity count by age groups among people with and without obesity

Comorbidity Count*	18–39 Years		40–64 Years		≥ 65 Years	
	Mean	SD	Mean	SD	Mean	SD
Obesity group	1.72	1.57	3.43	2.01	5.28	2.10
Non-obesity group	0.63	0.91	1.81	1.69	3.87	2.14
Obesity: Non-obesity group (relative ratio of number of comorbidities)	2.73		1.90		1.36	

*SD* standard deviation

Significant (*p* < 0.0001) differences in obesity versus non-obesity were observed in all the groups: 18–39 years, 40–64 years, ≥ 65 years

*Obesity was not included in the total comorbidity count

## Discussion

This claims database study confirmed that people diagnosed with obesity, as per the ICD-10-CM diagnoses code, had more comorbidities than those diagnosed without obesity. This was true for all age groups; however, this phenomenon was most pronounced in the youngest age group. Compared to other age groups, people with obesity in the youngest age group had a greater comorbidity burden, with a higher prevalence of most evaluated conditions. Furthermore, it is conceivable that the early onset of hypertension, dyslipidemia, and glycemia disorder observed in the younger age group with obesity could be a potential driver for the higher prevalence of coronary heart diseases and heart failure observed in the older age groups.

Our study findings were consistent with previous findings that have reported a higher prevalence of comorbidities in people with obesity compared to those without obesity, particularly cardiometabolic disorders [15–17]. Data from the National Health and Nutrition Examination Survey (NHANES, 2017–2018) showed that the prevalence of hypertension in people with obesity (61.0%) was nearly two times higher as compared to those with normal weight (33.1%) [18]. People with obesity also have a substantially higher prevalence of prediabetes and dyslipidemia [14, 16]. Nearly 30–53% of diabetes mellitus cases can be attributed to obesity in the US [19, 20]. In addition to cardiometabolic risk factors, obesity is linked to a higher incidence of cardiovascular events [21]. Severe obesity (versus normal weight) was linked to a nearly four-fold higher risk of heart failure and two-fold higher risks of coronary heart diseases and stroke [21]. Notably, the degree of obesity is associated with complex multimorbidity, with increased BMI increasing the risk of cardiometabolic multimorbidity [22]. Compared with normal weight individuals, the risk of developing cardiometabolic multimorbidity (presence of at least two from type 2 diabetes, coronary heart disease, and stroke) was almost five times higher for individuals with class I obesity (BMI = 30.0–34.9 kg/m^2^), and almost 15 times higher for individuals with classes II and III obesity (severe) combined (BMI = ≥ 35.0 kg/m^2^) [22]. Severe obesity is associated with an increased risk of cardiovascular mortality [23, 24]. The reported associations between obesity and the different cardiometabolic diseases suggest that obesity is an important target for cardiovascular disease prevention. However, on the contrary, we observed a lower prevalence of osteoporosis in people with obesity in ≥ 65 years age group. Similarly, in a cross-sectional study, women without obesity aged > 50 years were reported to be at an increased risk of osteoporosis [25]. In another study, higher BMI was positively linked to increased bone density, which may be attributable to the mechanical effect of body weight on osteocytes and osteoblastic differentiation [26, 27]. Under biomechanical stress, changes in biochemical markers have been observed in people with obesity, including a reduction in bone resorption markers that is greater than that observed for bone formation [28].

Our study focused on the younger age group with obesity and observed substantially higher comorbidities of hypertension, dyslipidemia, and prediabetes compared to those without obesity. This indicates an early onset of multiple chronic cardiovascular risk factors with obesity that can have a significant long-term impact. Furthermore, the high prevalence of these disorders in the younger age obesity group suggests many individuals likely had metabolic syndrome from an early age. Cumulative exposures to such risk factors at an early age can cascade to increased cardiovascular risk in later life, independent of later adult exposures [12, 29, 30]. Previous research has suggested that the association between obesity and cardiometabolic risk factors was stronger in younger individuals, putting them at an elevated risk for premature cardiovascular disease and heart failure [31, 32]. Interestingly, the risk of complex multimorbidity was found to be greater in people with obesity at younger ages (< 50 years) than those in the older groups [33]. This is consistent with our findings, which showed a higher relative ratio of comorbidities in the younger age group than in the older age group. Our findings emphasize the urgent need for early prevention of obesity and maintenance of normal weight throughout life to alleviate the burden of future cardiovascular events.

Individuals with obesity in our study exhibited a high prevalence of depression, which declined from the younger to the older age group. Consistent with our results, prior research has demonstrated an association between high BMI and risk of depression; people with obesity were 32% more likely to have depression compared with those with a healthy BMI of 18.5 to 24.9 kg/m^2^ [34, 35]. A meta-analysis also reported that 55% of individuals with obesity were likely to suffer from depression during their lifetime [36]. Another longitudinal international study reported that the higher the BMI, the higher is the risk of experiencing depressive symptoms [37]. Similar to our study, previous research suggests that osteoarthritis and obstructive sleep apnea are other prominent comorbidities in people with obesity [33, 38]. Notably, the odds for both obstructive sleep apnea and osteoarthritis increased significantly with the severity of obesity in this study, highlighting the importance of preventing further weight gain in individuals with obesity. Given the considerable burden of obesity-related disorders, the implementation of holistic treatment strategies is critical for the effective management of individuals with obesity.

Our study was based on a cross-sectional sample of an insured population from a health insurance claims database. As a cross-sectional study, it does not capture changes in obesity status or progression of related disorders. As a claims data study, it relied only on the diagnoses information to identify the prevalence of disorders. The database did not include actual patient weight or BMI but captured coded diagnoses. Another limitation of our study is the usage of ICD-10 codes for the diagnosis of obesity, which can vary in specificity. This variability may lead to inconsistencies in how obesity is recorded and interpreted across different healthcare settings. It is likely that obesity is under-diagnosed and under-coded in the claims databases. Such misclassifications would have resulted in differences and incomplete representation in the prevalence of obesity-related comorbidities between the groups in the current study. Furthermore, it can affect healthcare outcomes by underestimating the burden of obesity-related conditions, leading to insufficient resource allocation and suboptimal patient care strategies. Standardizing coding practices, enhancing training for healthcare providers and coders, and implementing more robust documentation requirements are recommended. Addressing the problems with under-diagnosis and under-coding of obesity is crucial for improving the accuracy of claims data, enhancing healthcare outcomes, and ensuring fair reimbursement practices.

The absence of social determinants of health data such as socioeconomic status, education, neighbourhood and physical environment, employment, and social support network data in our analysis may limit the comprehensiveness of our findings and the ability to fully understand the root causes of obesity and related comorbidities within the studied population. Future research should consider incorporating these factors to provide a more holistic view of the factors contributing to obesity and to design more targeted and equitable interventions. Additionally, the study included only people insured by employer-provided Commercial and Medicare plans; therefore, results may not be generalizable to Medicaid, uninsured people, or those covered by other plans. Future research should also consider using additional data sources, such as electronic health records and patient self-reports, to complement ICD-10 codes and claims data. This multi-faceted approach may help capture a more accurate picture of obesity prevalence and its impact.

## Conclusions

This Medicare and Commercial healthcare claims study in the US identified a range of comorbidities in people with and without obesity in their healthcare settings and provided insights on how their prevalence differs across different age groups. People diagnosed with obesity had higher prevalence of comorbid disorders than those without obesity, most notably with hypertension, and dyslipidemia, with increasing age. The younger age group with obesity had a high prevalence of cardiometabolic comorbidities, suggesting potential risk for early onset of cardiovascular events in later life, which may lead to greater individual and societal burden. Knowledge of prevalent disorders in people with obesity can help healthcare professionals better identify patient needs in the different age groups, prevent long-term adverse health outcomes, and facilitate judicious decision-making by policy makers and payers.

## Data Availability

Data for these analyses come from a proprietary database, the Merative™ MarketScan^®^ Research Databases and are not publicly available. The source data (administrative claims) analyzed during the current study are available from the data owner (Merative™ MarketScan^®^) for a fee. Research outputs are available from the corresponding author on reasonable request.

## Abbreviations

BMI: Body mass index
CHD: Coronary heart diseases
US: United States
ICD-10-CM: International Classification of Diseases, 10th Revision, Clinical Modification
HIPAA: Health Insurance Portability and Accountability Act
SD: Standard deviation
NHANES: National Health and Nutrition Examination Survey
MASLD: Metabolic dysfunction-associated steatotic liver disease
MASH: Metabolic dysfunction-associated steatohepatitis
